# Internationalising Health

**Published:** 1923-09

**Authors:** 


### INTERNATIONALISING HEALTH.

### WORK OF THE ROCKEFELLER FOUNDATION.

UNDER the title of "The Rockefeller Foundation," Mr. George E. Vincent, the President of the Foundation, has issued a combined review of the year 1922 and a summary of the first decade's work. It forms a chronicle of achievement remarkable enough to justify the belief that the Foundation will go far to revolutionise the world's ideas about preventive medicine. Money has been poured out lavishly. Last year more than three millions sterling were spent, and the expenditure of the decade has exceeded fifteen millions in English money, and definite results are not incommensurate with the effort. Yellow fever is being driven from the world so successfully that the comparatively small number of reported cases of the disease last year were confined to Mexico, to a restricted area in Northern Brazil, to points on the West Coast of Africa, and to ships *en route* from one of these countries; and there is a highly significant map of the Western Hemisphere showing the steady reduction of infected areas.

### Attacking the Hookworm.

Encouraging success has attended attempts to control malaria, while a widespread system of activity has been created for dealing with hookworm disease, a malady prevalent throughout areas inhabited by nine hundred millions of people. The report points out that:—

The control of hookworm infection is an effective means of educating people in the meaning of public health work and of persuading them to support more comprehensive measures for preventing of other diseases as well. In 1922 the International Health Board had a part in hookworm control activities in 22 governmental areas in the United States, the West Indies, Central America, South America and the Far East. Since 1911 the Board has co-operated in 69 states and countries. In 54 control measures were carried out; in 15 others only surveys were made. The policy of the Board in this work has been to undertake control measures only on the invitation of a government which bore from the first at least a small part of the expense, and agreed to take on each year an increasing proportion of the cost until it finally assumed entire responsibility for the continuance of the project. In order to measure the progress of control the Board in the last three years made re-examinations of school children in 66 counties in the Southern States and compared results with those of the original surveys, which were made between 1910 and 1914. On the average a reduction of 47.5 per cent. was shown.

### The London School of Hygiene.

The International Health Board has from the beginning been so much impressed with the need for trained sanitarians that it has welcomed opportunities to co-operate in establishing schools of public health:—

During 1921 a request was received from the British Ministry of Health to share in the creation of such a training centre in London. Negotiations resulted in an agreement by the International Health Board of the Rockefeller Foundation to give $2,000,000 for land, building and equipment for a school which the British Government has undertaken to maintain. A site on Gower Street near the British Museum and University College Medical School has been purchased, and a committee is at work upon a scheme of organisation and plans for a building. This School of Hygiene in London will occupy a strategic position. For teaching purposes it will command the great scientific resources, public health records and the well-trained, experienced personnel of the unusually efficient health services of Great Britain. By virtue of the position in the British capital the new institution will exert an influence throughout the Empire. It seems likely also to serve as a training centre for prospective health officers from many other nations.

### Promoting International Hygiene.

The scientific basis of preventive medicine has long been recognised as a matter of international concern:—

The first European Conference to consider health problems was held in 1851. Twelve nations were represented. Concerted measures against cholera, plague and yellow fever were adopted. Thereafter at intervals of a few years other congresses were called to insure better teamwork in preventive medicine. In 1902 an International Sanitary Bureau was established in Washington by the Pan-American Union. Finally, in 1908, a permanent International Office of Hygiene was established in Paris under the auspices of thirteen nations. Voluntary associations have also a part in this common battle against disease. The League of Red Cross Societies, with headquarters in Paris, includes public health activities in its international programme. The medical congresses already mentioned have had an important bearing on the spread of public health knowledge and practice. The work of the Rockefeller Foundation, which is described in this review, lies largely in the field of international hygiene. The most significant development in this movement is the recent creation under the League of Nations of a Health Organisation which has the direct support of fifty-two nations and the sympathetic co-operation of the United States. The new body has reached a working agreement with the International Office of Hygiene in Paris and will doubtless have cordial relations with the International Sanitary Bureau. The programme of the League's Health Organisation includes the gathering of vital statistics, prompt notification of epidemics, standardising of vaccines and sera, international conferences and exchanges of health officers, securing of better health conditions for sailors on shipboard and in ports, co-operation with League mandatories, with the Commission on Opium and with the International Labour Office. The International Health Board has made appropriations to the League of $344,440, to be used over a period of three to five years in a demonstration of the feasibility and value of an international epidemiological service and an international exchange of health officers.

### Advanced Students from Twenty-three Countries.

Mention is made in the report of the Fellowships in public health, medical education and nursing by means of which the various agencies of the Rockefeller Foundation are providing advanced training for men and women who are preparing themselves for careers in these fields:—

During 1922 the total number of Fellowship holders was 237. Of these 164 were appointed directly through departments of the Foundation, while 73 were selected and supervised by special committees of the National Health Research Council. The list does not include 105 fellows studying at the Peking Union Medical College, nor bourses for 157 health visitors in France and for 7 French nurses in London hospitals. From its inception in 1915 to December 31, 1922, 431 fellows have studied under this fellowship plan. The fellowship policy of the Foundation aims at flexibility, selection and specific preparation. No fixed number of fellowships is assigned to any one subject or country. Only candidates of exceptional promise are chosen, to whom positions in Government or institutional service have been assured on the completion of their studies.

